# On the Illumination Influence for Object Learning on Robot Companions

**DOI:** 10.3389/frobt.2019.00154

**Published:** 2020-01-21

**Authors:** Ingo Keller, Katrin S. Lohan

**Affiliations:** ^1^Department of Mathematical and Computer Science, Heriot-Watt University, Edinburgh, United Kingdom; ^2^EMS Institute for Development of Mechatronic Systems, NTB University of Applied Sciences in Technology, Buchs, Switzerland

**Keywords:** object recognition, object learning, visual perception, data augmentation, human-robot interaction, long-term engagement

## Abstract

Most collaborative tasks require interaction with everyday objects (e.g., utensils while cooking). Thus, robots must perceive everyday objects in an effective and efficient way. This highlights the necessity of understanding environmental factors and their impact on visual perception, such as illumination changes throughout the day on robotic systems in the real world. In object recognition, two of these factors are changes due to illumination of the scene and differences in the sensors capturing it. In this paper, we will present data augmentations for object recognition that enhance a deep learning architecture. We will show how simple linear and non-linear illumination models and feature concatenation can be used to improve deep learning-based approaches. The aim of this work is to allow for more realistic Human-Robot Interaction scenarios with a small amount of training data in combination with incremental interactive object learning. This will benefit the interaction with the robot to maximize object learning for long-term and location-independent learning in unshaped environments. With our model-based analysis, we showed that changes in illumination affect recognition approaches that use Deep Convolutional Neural Network to encode features for object recognition. Using data augmentation, we were able to show that such a system can be modified toward a more robust recognition without retraining the network. Additionally, we have shown that using simple brightness change models can help to improve the recognition across all training set sizes.

## 1. Introduction

Using robotic companions in unconstrained, domestic environments poses new challenges to the task of object recognition and learning. In this work, we focus on such use-cases in which one cannot draw from a large set of images for training since the presentation of particular objects might occur infrequently and only over short periods. Moreover, consumer-oriented robotic hardware usually does not allow for computationally expensive training (e.g., state-of-the-art deep learning networks). The learning needs to be fast enough, in the range of seconds rather than hours, to be of value for the user. Additionally, the objects that will have to be learned are not necessarily known upfront and might only get presented over time rather than all at once, which limits the possibility of pre-training networks.

New developments in deep learning, computer vision and robotics research together with the availability of highly integrated, powerful mobile computer systems has made it possible to create robotic systems that can operate in private households. Interactive household robots have started to appear in consumer stores, such as the Jibo^1^, the Buddy Robot^2^, or the robotic kitchen^3^. Also, research is increasingly focusing on providing assistance and companion robots for socially supporting children or the elderly. All of these examples will lead to the availability of personal robotic assistants in the near future, the research of long-term engagement with such systems is still in its infancy. A major obstacle for interaction is the real-world environment since it is less controllable than a laboratory and therefore presents new challenges to state-of-the-art approaches. The ability to cope with changes in the environment will be an important factor in the acceptance of interactive robotic systems.

In Human-Robot Interaction (HRI), triadic interactions (Imai et al., 2003) are one of the most commonly studied problems to create natural interaction between robots and humans. To jointly manipulate objects, Moldovan et al. (2012) first requires the robotic systems to recognize them reliably. Here, we focus on improving object recognition in noisy environments to learn to cope with real-world constraints. In particular, we research the influence of illumination changes and their impact on object recognition in the context of real-time capable recognition systems without prior knowledge.

One state-of-the-art visual recognition systems for incremental interactive object learning is provided by the iCub community. The approach utilizes a combination of a Deep Convolutional Neural Network (DCNN) for feature generation with a Multiclass Support Vector Machine (SVM) for classification of objects that were shown to the iCub (Pasquale et al., 2015a). An exhaustive evaluation of the performance of the combined networks can be found in Sharif Razavian et al. (2014). The system provides a method for long-term object learning due to its incremental training on images which are acquired by interaction with the robot. Classifiers can be trained near real-time and are usable in real-world scenarios in which novel objects can appear at any time.

Training each robot individually on the objects of its particular environment is inefficient and doesn't scale. Therefore, pre-trained robots that can expand their knowledge on-the-fly are required for the real world. Hence, we looked into the re-usability of existing datasets for training classification models following the *off-the-shelf* approach for feature generation of Sharif Razavian et al. (2014). Reusing feature generation models across different robotic platforms would dramatically reduce computational requirements and is preferred over training individual robots. Therefore, we focus on global illumination changes that might occur due to light changes throughout the day, different viewpoints or sensors used. As indicated by our previous work (Keller and Lohan, 2016), light changes have a negative impact on state-of-the-art object recognition.

The contribution of this work is a systematic analysis of the impact of light changes on using two different light models over a wide range of parameters. The benefits of data augmentation are analyzed for the mentioned feature generation and classification methods. Both methods are treated as black-box systems to provide a baseline for further research of methods for illumination robustness in systems with low computational resources for extensive retraining, such as the mentioned robot companion systems.

In particular, we research recognition capabilities under the assumption of a low number of input images corresponding to short interaction durations. While the datasets used were created throughout multiple training sessions in the lab, in real-world scenarios, this amount of time to acquire the data might not be available. Therefore, we want to increase the recognition performance in circumstances that provide only a small set of training examples. As will be discussed in 3, we used the iCubWorld28 dataset to identify the impact of illumination changes on the object recognition pipeline. With the larger iCubWorld Transformation dataset, we transferred and tested our method to a broader diversity of object manipulations, background changes, and brightness variance.

## 2. Background

### 2.1. Visual Features

Two approaches for feature generation on 2D images can be distinguished in state-of-the-art object recognition. Methods, such as SIFT, SURF, and ORB, use local, keypoint-based feature sets that are generated from template images. These features are robust against a variety of transformations, including scaling and rotation. Recognition of 3D objects can then be achieved by creating object feature databases based on different viewpoints (Yu et al., 2014) and using matching techniques, such as RANSAC (Fischler and Bolles, 1981) to determine if a given object's feature set can be considered a model for a set of features found in a test image. While these approaches result in robust recognition and are therefore widely used, they suffer from an increase in computational cost in the cases of a high number of objects or high resolution of images.

Another type of object recognition method utilizes Deep Learning to generate features using pre-trained networks (Sharif Razavian et al., 2014; Fischer et al., 2016). Networks, such as the AlexNet (Krizhevsky et al., 2012) are trained on large image datasets and, once trained, can be treated as black-box filters that generate features from images. The benefit of this technique is that the resulting feature vectors are of fixed-length, which allows for the use of standard classification methods, such as SVMs.

We investigate the latter approach since it is used in a real object recognition pipeline for incremental learning on an existing humanoid robot platform (iCub) and due to the availability of datasets with many different objects which were captured during interactions with the robot. The next part of this section will give an overview of the background of illumination variations.

### 2.2. Illumination Variations

Dealing with illumination variations is a long-standing problem for visual recognition systems, especially for the perception of color. Ever since Land (1964) introduced the Mondrian experiment and proposed his Retinex model, it has become clear that the human visual system perceives the color of an object not only based on its photometric properties. The lighting condition of the surrounding environment is taken into account by our brains to tune our sense to perceive a certain color, even if it is not physically present in the scene. This problem is known as color constancy and has been addressed by computer vision research in many ways. A comprehensive overview can be found in Foster (2011). Furthermore, the perceived color of an object can vary between people, and it is thought to depend on people's age, gender, and general light exposure habits (early birds vs. night owls) (Lafer-Sousa et al., 2015). These findings indicate that the human visual system is capable of adapting over time and learning certain illumination scenarios, which it uses as priors to adjust the perception of color.

To capture this ability of the human brain, a wide range of methods have been proposed to tackle robustness against illumination changes. However, methods that aim to take light variations into account either suffer from a high computational cost, which renders them infeasible to use in real-time systems with limited computational resources, or make assumptions that are not met by generic, natural images. The first case is presented by state-of-the-art methods for object recognition, such as the currently fastest object detector, YOLOv3 (Redmon and Farhadi, 2018). Deep Learning methods can achieve a high performance but require the support of powerful GPUs which are usually not available on the type of robot systems we are targeting (see Reyes et al., 2018). The second class of methods can be found in more specific areas. A prominent example is face recognition, in which most illumination models make assumptions that do not hold for general objects, such as Lambertian surface reflectance, underlying facial models, or more generally the importance of facial landmarks over general object features (e.g., Le and Kakadiaris, 2019).

While state-of-the-art methods have advanced the standard for illumination robustness, they are also usually computationally expensive. Using pre-trained deep learning networks has become feasible using onboard computing units but retraining them, especially in an incremental and interactive way, is not yet possible. In the next section, we are introducing a visual pipeline that is capable of handling such constraints (real-time capability, incremental/interactive object learning, and low amount of training data) and describe our approach to improve it afterwards.

### 2.3. Visual Recognition System

**Figure 1:**
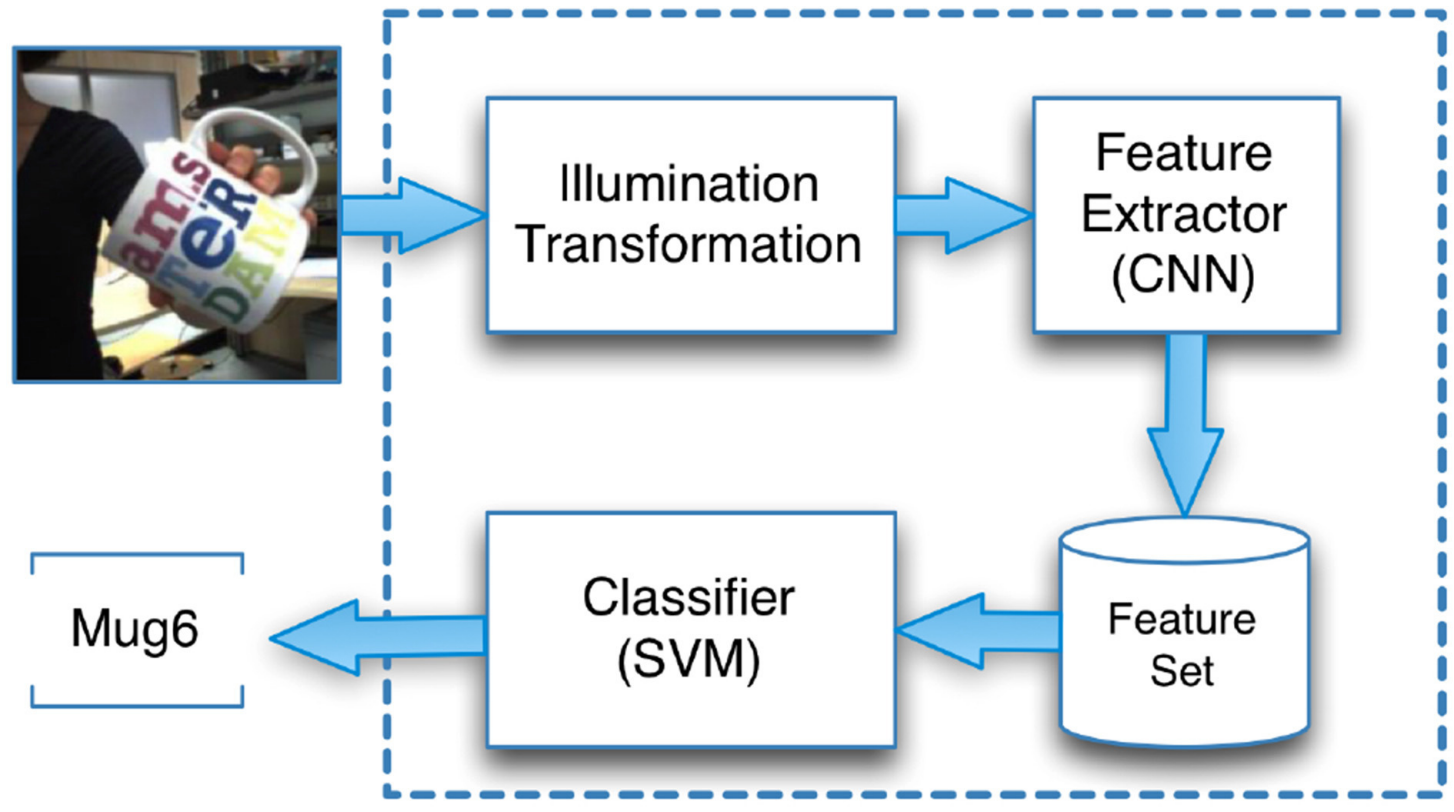
Our adaptation of the iCub's recognition workflow includes the illumination transformations.

The Interactive Object Learning (IOL) system^4^ is part of the open source software stack for the iCub robot^5^. Here, we focused on the Feature Extractor and Classifier^6^ (Figure 1).

The Feature Extractor is based on the *BLVC Reference CaffeNet*, which is provided by the Caffe library (Jia et al., 2014). This network was trained on the ImageNet dataset (Krizhevsky et al., 2012), which contained more than 1 million high-resolution images from 1,000 object categories. The Feature Extractor generates a feature vector that is characteristic for the input image under a given DCNN. The vector corresponds to the vector representation of the highest convolution layer of the DCNN (Pasquale et al., 2015b). A Multiclass Linear SVM was used for classification of feature vectors. The SVM is trained with a one-vs-all strategy for 1,000 epochs and thus provides a classifier for each object.

To use the given implementation without the robot involved, we had to separate the recognition workflow from the rest of the system to use the pipeline in a standalone manner. The separation was necessary to provide an analysis environment that resembles the original system as closely as possible (see Pasquale et al., 2015b). Since the provided solution is a highly integrated system, we had to rearrange the workflow to fit our needs. This way, we also decreased the processing time for the analysis. Due to performance issues, we replaced the *linearClassifier* module from the pipeline with the *LinearSVC* implementation from the sci-kit package (Pedregosa et al., 2011) after ensuring that we achieve comparable results. All feature vectors were generated upfront as we did not require human interaction for our experiments.

### 2.4. Data Augmentation

Data augmentation or preprocessing is a way for recognition methods to enhance input signals and to make the recognition more robust against known transformations. It is a standard tool for image recognition.

A wide variety of data augmentations have been used to capture different types of invariance, such as translation, rotation, mirroring, distortions, color, and light changes. Bhattacharyya (2011) provided a brief overview of additional color image preprocessing techniques. Ahmad et al. (2017) use detexturized, decolorized, edge enhanced, salient edge map based, and flip/rotate images to improve DCNN-based recognition in visual searches. More specialized versions of preprocessing are available if targeted tasks (e.g., in face recognition) can be narrowed down and underlying information can be modeled more precisely (see Zou et al., 2007; Han et al., 2013).

For general object recognition, computationally inexpensive augmentations that handle light changes are limited and usually involve at least gamma and brightness corrections. For example, Fischer et al. (2016) augment training samples with a gamma adjustment (−0.5, 0.1) and a brightness adjustment (−0.2, 0.2). Dosovitskiy et al. (2015) chose a gamma value between 0.7 and 1.5, incorporated an additive brightness with Gaussian augmentation and a contrast modification. Kim et al. (2019) use brightness and contrast in low-illumination scenarios for video surveillance systems. Howard (2013) uses randomly changed contrast, brightness, color, and random lighting noise to capture light change variance. They base their modifications on Krizhevsky et al. (2012), which also provides the DCNN used in our work and should therefore already capture some variance. However, as we will demonstrate, there is still room for improvement.

So far, we discussed augmentations in data space, which are well-established techniques. To a lesser degree, augmentation methods in the feature space are explored. To understand data augmentation for classification, Wong et al. (2016) used warping in the feature space to improve recognition on the MNIST dataset. For our approach, we take the idea of image concatenation (e.g., Saitoh et al., 2017), apply it as feature concatenation and combine it with gamma and brightness modification.

While widely used, to the best of our knowledge, no systematic analysis of the impact of gamma and brightness changes for object recognition have been conducted. The parameters of these modifications used in the literature vary and their separate effects on the recognition are not determinable as they are usually mixed with other types of augmentations and are not reported separately. In our work, we want to provide a starting point for a more systematic analysis. Also, we did not incorporate light modification models, which come with a too high computational cost for online learning, such as Gabor filters as suggested by Welke et al. (2006). Additionally, we will demonstrate that even though a DCNN was trained taking light change augmentations into account, it still can benefit if used as a black-box feature generator. Important to note is that we are not trying to enhance the feature generator itself as this would involve computationally expensive training. Instead, we are aiming to generate enhanced feature vectors to help the classification step under the assumption that creating multiple features from the images can be done in parallel and therefore do not add much to the computation time for the feature generation.

## 3. Datasets and Methods

In this section, we describe the datasets, the models and the experiments parameters. As mentioned, we are focusing on global illumination changes as we consider them to be of greater importance than local ones. While local illumination changes, such as reflection or shadow can have a high negative impact on the recognition as well, in the HRI scenario that we are addressing they are not necessarily persistent across multiple images during an interaction due to the changing orientation of the objects. Thus, local illumination changes are not considered in this paper.

The experiments are an extension of our earlier work which can be found in Keller and Lohan (2016). In our previous work, we used a small image dataset in combination with two illumination models to simulate linear and non-linear brightness changes to understand the impact of changes in light to the DCNN/SVM-based learning approach. In this paper, we compare the effect on a larger dataset with higher variability in object presentations as well as present a method to make use of the findings to improve the recognition process itself.

While a practical experiment could have been conducted to show the method's behavior under different illumination conditions, we chose to start with modified sample images of datasets for repeatability. This way, we are also able to look separately at linear and non-linear changes while this is much more difficult to achieve in an experimental setup. Both types of changes might occur at the same time in an experimental setup in a way that is not trivial to control. For example, a robot might use consumer camera sensors with a Automatic Gain Control (AGC) that influence the resulting image in a non-linear manner when the surrounding illumination is changed (Fowler, 2004). Due to the integration of current image sensors themselves, it can be impossible to deactivate these assistance systems. Linear changes might occur when blinds are used in different positions, limiting the amount of light from the outside.

### 3.1. DS1—iCubWorld28

The first dataset is the iCubWorld28 dataset from Pasquale et al. (2015a) referred to as *DS1*. It represents the visual perception of the iCub. It was created during a 4-days interactive session. It consists of nearly 40,000 images of 28 objects distributed over 7 object classes with more than 1,300 images per object.

The dataset comes separated by day and is split into a training and test set per day. Since we were interested in the overall performance of the approach, we merged all images per object into one set as this gives the wide range of original illumination changes and allows for analyzing the sample size dependency (see 4.1). From the merged sets a number of training and test sets were randomly selected. First, the 400 images for four test sets were chosen. Afterward, images for the different sized training sets were selected. Thus, the results of the corresponding training and test sets are comparable to each other and are based on balanced sets for all objects.

### 3.2. DS2—iCubWorld Transformation

The second dataset is the iCubWorld Transformation (Pasquale et al., 2016) referred to as *DS2*. The dataset consists of more than 600,000 images with at least 3,000 images per object. It provides five different types of visual transformations; 2D, in-plane and 3D free rotations to provide the robot with different viewpoints of the object, scaling transformation in which the human moves the object either closer or further from the robot's position and a transformation in which the human is moving in a circle around the robot to change the background while keeping approximately the same distance from the robot. Additionally, a mixed transformation was included in which the object was presented in a free moving manner. All objects are captured during two different days each. The dataset contains image sequences from 15 object categories with 10 objects in each category giving a total of 150 objects (IROS 2016 subset from Pasquale et al., 2016).

We chose this dataset as it contains more variability in the presentation of the objects (e.g., a wider range of illumination changes) and also contains more objects (150 vs. 28). This way we can test our method on a more challenging task but can compare the results as the acquisition of the second dataset was similar to the first. The preparation of the second dataset follows the method of the first one.

### 3.3. Illumination Change Models

**Figure 2:**
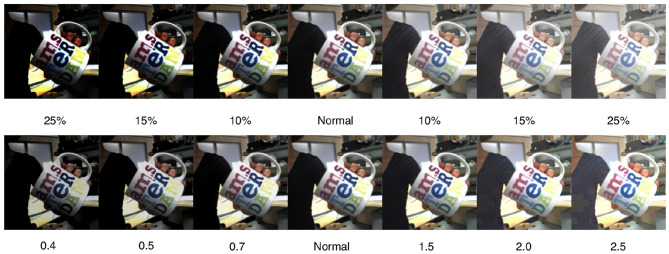
The unaltered sample (middle) and its transformations toward the extreme values: (top) linear model—example value changes in HSV color space with back-transformation into RGB color space, (bottom) non-linear model—example gamma value changes in RGB color space.

To account for linear and non-linear light changes, we generated new images from the dataset. We chose the value modification from the HSV color space as an example of a linear model. HSV stands for *hue, saturation*, and *value*, where *value* accounts for brightness. The second model is given by the gamma transformation which serves us as a non-linear modification. The transformation was done using OpenCV (Bradski, 2000). Figure 2 shows examples for both modifications. While these changes seem to be easy for the human eye they have an impact on recognition systems that operate on pixel values.

#### 3.3.1. Linear Model

The images were transformed into the HSV color space for modification. This allows for changes to the luminance without interfering with the colors and is one of the common color spaces for classical visual recognition. The model is defined as *V*_*out*_ = *V*_*in*_ ± (*V*_max_*V_*c*_). After the color space transformation *V*_*c*_ was changed to 5, 10, 15, and 25% for both lighter and darker appearance. For the recognition task, the images are transformed back into RGB color space before being fed to the DCNN.

#### 3.3.2. Non-linear Model

The gamma correction is a non-linear transformation that is often used to enhance the visual appearance of images that are under- or overexposed. It is defined as $V_{out}=V_{in}^{1/\gamma }$. For the γ we chose 0.4, 0.5, 0.7 for darker and 1.5, 2.0, 2.5 for brighter images.

#### 3.3.3. Parameter Selection

The choice of the parameter values is based on selected representatives for non-trivial changes. If the transformation effect gets much stronger differences in color can degrade toward black or white areas destroying the included color information and leading to unrecognizable images. While this is an effect that image recognition approaches have to deal with, it is not the focus of the paper in which we want to improve recognition under different lighting conditions while being in a reasonably well-lit environment. Table 1 gives an overview of the parameter sets we chose and how they are named in our paper.

**Table 1:** Naming convention for the modified sets.

**Names**	**Sets**
**LINEAR CONDITION**	
0	*V* = 0
-25:1:25	*V* ∈ {−25%, 0, 25%}
**NON-LINEAR CONDITION**	
1	γ = 1
0.4:1:2.5	γ ∈ {0.4, 1, 2.5}
**COMBINED CONDITION**	
Mix	γ ∈ {0.4, 2.5} and
	*V* ∈ {−25%, 25%} and
	1 set of unmodified images

#### 3.3.4. Measurement

For our experiments, we report the average accuracy. All reported accuracies are an average over a 4-fold experiment run. Minimal and maximal errors can be found in the diagrams but are not reported for a clearer presentation of the findings. Due to their small variability, they did not have an impact on the conclusions. All experiments were supported by test sets with 400 samples per object.

## 4. Experiments and Results

### 4.1. Sample Size Dependency

**Figure 3:**
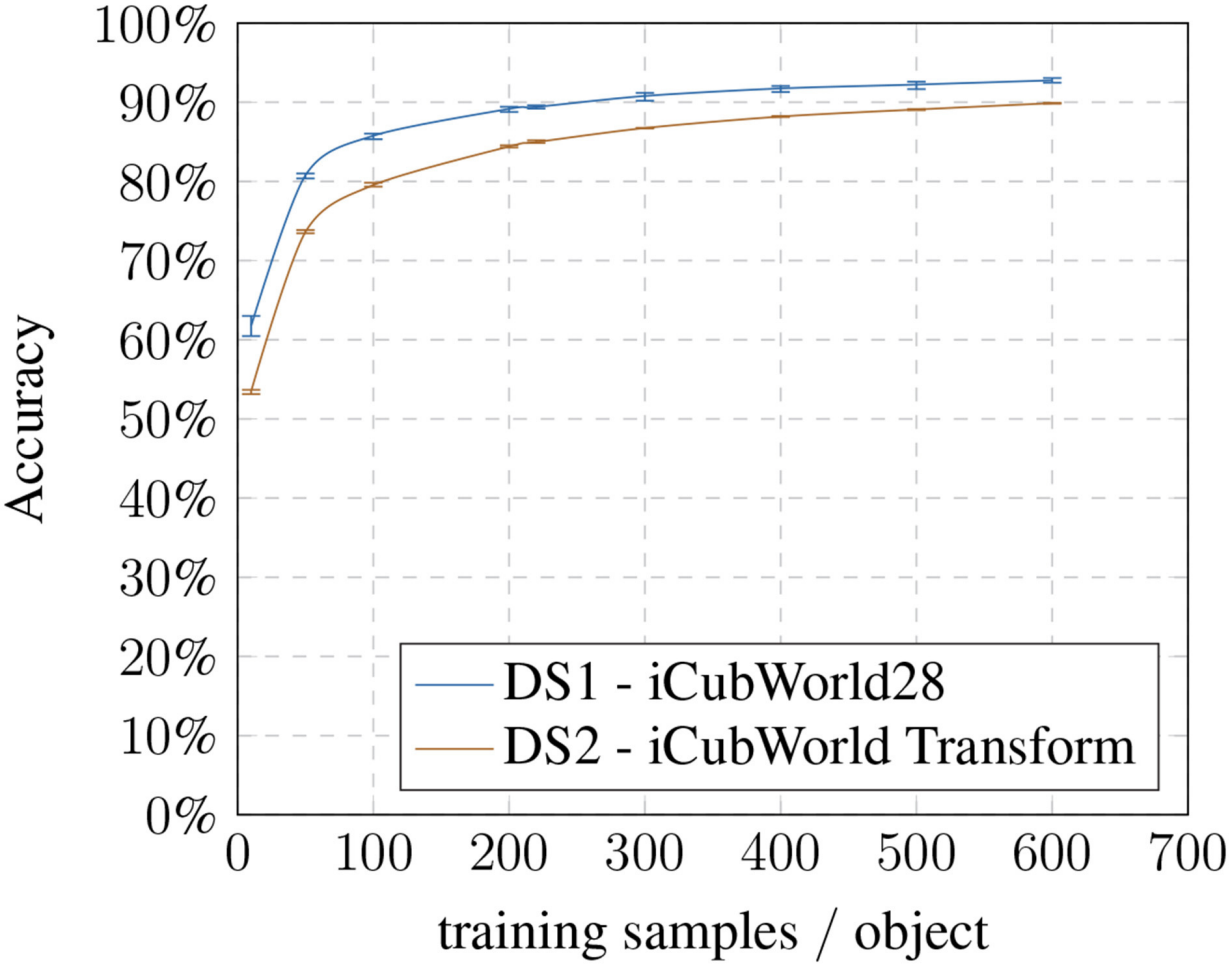
Baseline: accuracy based on training samples per object.

First, we established a baseline for the recognition task without any modifications to analyze the performance depending on the number of training samples used (Figure 3).

With 10 training samples, the method achieves an avg. accuracy of 61.74% going up to 92.77% for 600 images on the *DS1* (28 classes) and avg. accuracy ranging from 53.50% for 10 images up to 89.88% for 600 images on the *DS2* (150 classes). The avg. accuracy is highly stable over all runs with a variation of ±1.3% maximum in the worst case (10 training samples / *DS1*). The baseline results show that the gain in recognition performance using more than 100 training images becomes comparably smaller (Figure 3). As expected, the recognition pipeline performs similarly on both datasets and benefits from larger training set sizes in the beginning. The absolute difference between the datasets can be explained due to the second one containing more challenging presentations as well as more object classes.

**Figure 4:**
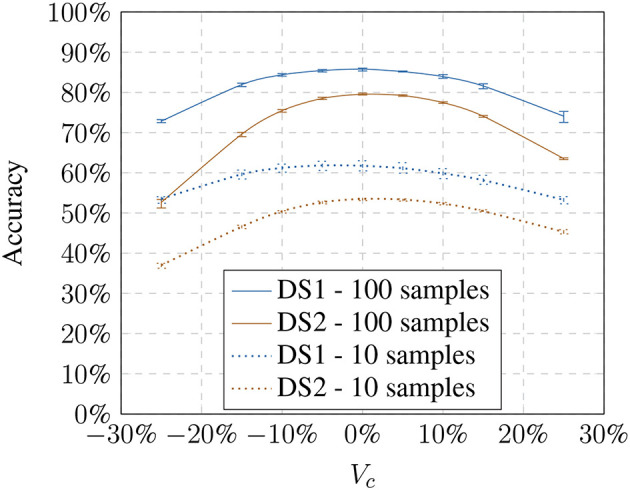
Influence of the linear model (*V*_*c*_) on the accuracy showing the loss toward stronger transformation values.

**Figure 5:**
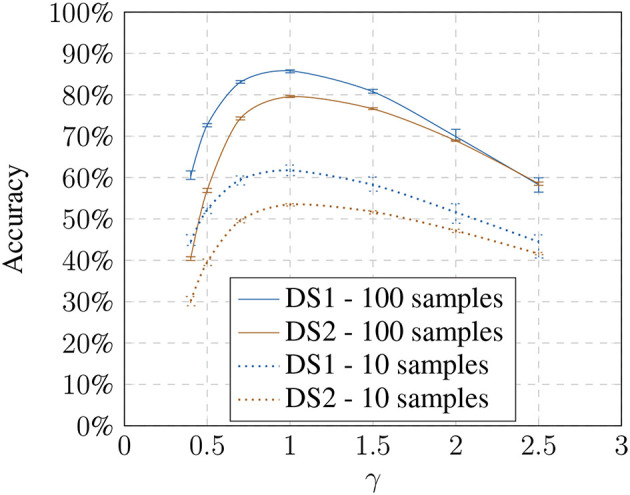
Influence of the non-linear model (γ) on the accuracy showing the loss toward stronger transformation values.

Next, we tested how the recognition rate changed if the visual pipeline has to recognize images with model-based altered brightness images. The baseline training was used and presented with altered test images based on the respective models (Figures 4, 5). The modified test image sets always contain only one modification to test the behavior on the modification limits. The results show that with an increase or decrease in brightness the recognition performs worse. The effect is consistent with Keller and Lohan (2016) although we used a random training and test sample selection this time.

Using a larger number of training samples seems preferable due to better recognition and thus compensating the effects of the illumination influence. However, it defeats the purpose of our approach which is meant to improve the recognition using small training samples sets. Under real-world constraints, interactions with the objects might only happen over a very brief period. Additionally, we had to consider that the training time of a 1-vs-all Multiclass-SVM increases quadratically with the number of classes. The training with 600 samples per object results in training on 16,800 feature vectors for *DS1* and 90,000 for *DS2* for each object classifier (28 for *DS1* and 150 for *DS2*). We chose to perform the following experiments on 100 training samples per object since our focus is on small training sets. However, in 4.3, we will show that the results are generalizable and independent of training set sizes.

### 4.2. Brightness Dependency

**Figure 6:**
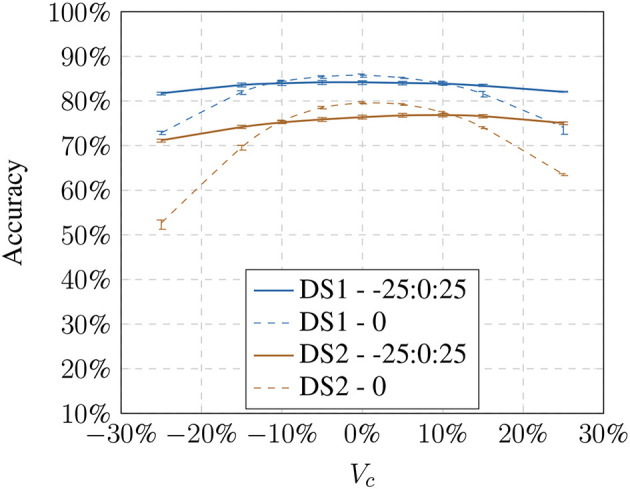
Influence of the brightness change: using linear modified training samples shows an increase in recognition accuracy toward the extreme values while having a decrease for non-modified images.

**Figure 7:**
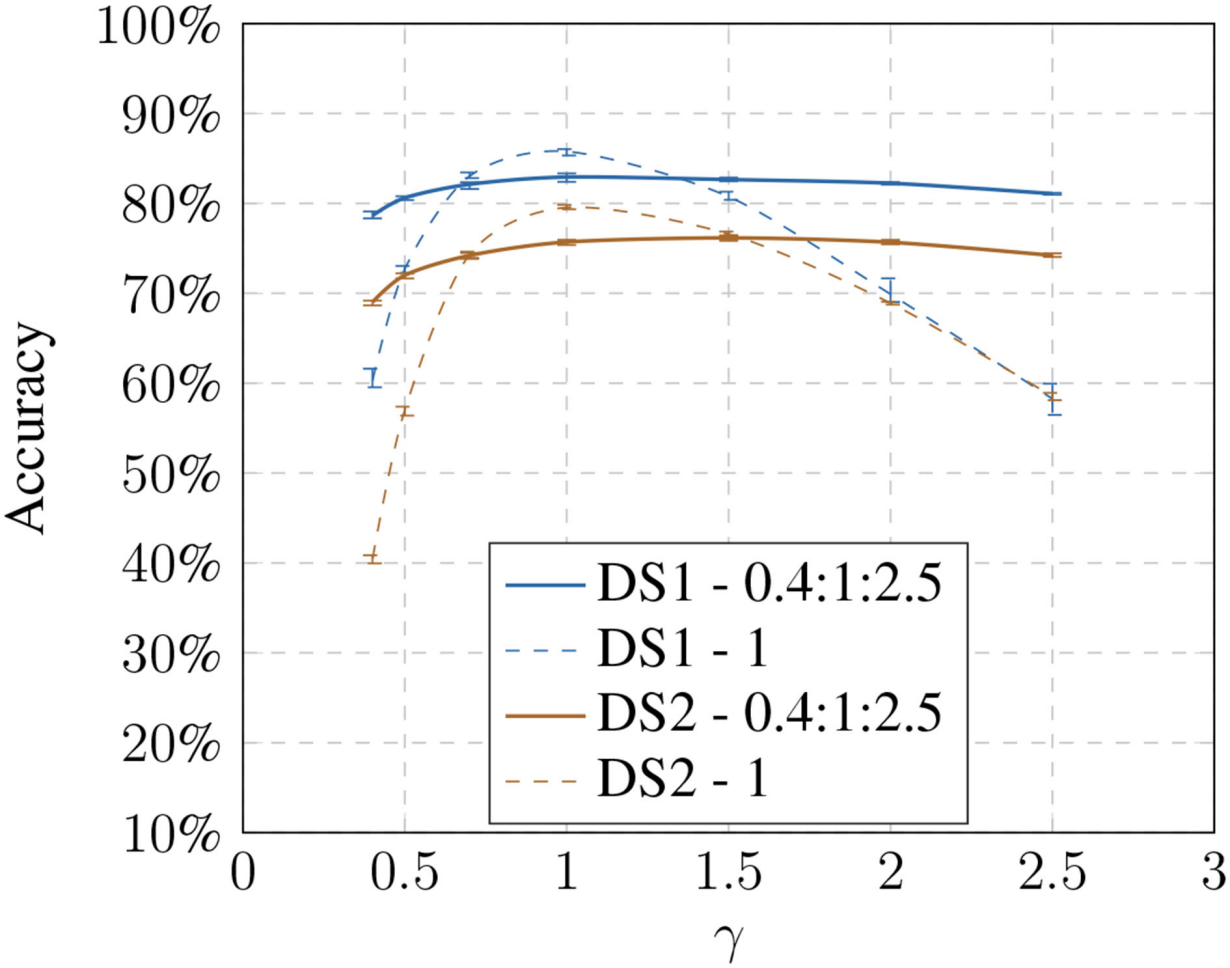
Influence of the gamma change: using non-linear modified training samples shows an increase in recognition accuracy toward the extreme values while having a decrease for non-modified images.

After identifying the influences of brightness on the recognition process, we modified the training set to include altered images and tested the trained system against modified test sets (see Figures 6, 7). In Keller and Lohan (2016), we showed that it is sufficient to include only the modified images with the most extreme changes. Adding the modified images, the size of the sets increase from 10 to 30 and from 100 to 300 images, respectively. In our previous work, we have shown that this is a sufficient approach for a comparison (see Keller and Lohan, 2016).

For both models, we can show that adding these images increases the avg. accuracy drastically; in the best case we could achieve an increase from 58.26% up to 81.08% on *DS1* and from 40.43% up to 69.01% on *DS2* under the non-linear model (see Table 2). Recognition against the baseline training decreases slightly, suggesting that there is a trade-off between generalization and specificity.

**Table 2:** Selected avg. accuracy values—modified training sets, 100 samples.

***DS1***			
Linear condition	−25%	Not modified	+25%
0	72.84%	85.77%	74.07%
−25:0:25	81.72%	84.18%	82.03%
Non-linear condition	0.4	Not modified	2.5
1	60.27%	85.77%	**58.26%**
0.4:1:2.5	78.64%	82.93%	**81.08%**
***DS2***			
Linear condition	−25%	Not modified	+25%
0	52.65%	79.54%	63.53%
−25:0:25	71.18%	76.37%	75.05%
Non-linear condition	0.4	Not modified	2.5
1	**40.43%**	79.54%	58.53%
0.4:1:2.5	**69.01%**	75.70%	74.21%

With our first set of experiments, we showed that the investigated visual recognition pipeline is indeed susceptible to variations in illumination. We also show that by using modified training samples, the adverse effect of light changes can be circumvented to some degree. While this improvement comes at the cost of a small drop in recognition in the non-modified condition the overall positive effect justifies this modification as the recognition shows a much better generalization behavior across model-based changes and hence can be considered more robust.

### 4.3. Feature Fusion

In the last set of experiments, we want to make use of these findings to increase the overall performance of the recognition. So far, our experiments used the approach of training *n* sample images and testing individually against independent *m* test samples and relied on data augmentation in the input space. However, since we know which modified images belong to each other, we can make use of that additional knowledge to further improve the recognition. Therefore, we employ data augmentation in the feature space to bind the original images with their modifications. All feature vectors corresponding to one original image and its augmented versions are concatenated and fed into the SVM; both for training and testing. Thus, the dimensionality of the search space for the SVM increases and captures different light changes within one data sample. For example, the *mix* condition contains two feature vectors for the linear model, two for the non-linear and the non-modified feature vector hence the resulting feature vector is five times bigger than the ones from our other previous experiments.

Table 3 shows the results of the experiment for 10 and 100 training samples. For both training set sizes, the recognition improved taking the model-based modifications into account. For 10 training samples per object an increase of the avg. accuracy from 61.74% (baseline vs. baseline) up to 65.28% (mix vs. mix) and for 100 samples an increase from 85.77 to 90.00% are found for *DS1*. *DS2* shows an improvement from 53.50% up to 57.53% for 10 training samples and from 79.54% up to 85.41% for 100 training samples.

**Table 3:** Fused feature recognition avg. accuracy.

**Training**\** set**	**Not**	**Linear**	**Non-linear**	
	**modified**	**−25:0:25**	**0.4:1:2.5**	**Mix**
***DS1*****—10 SAMPLES**				
Not modified	**61.74%**	60.68%	58.72%	59.40%
Linear	60.72%	63.79%	–	–
Non-linear	59.56%	–	65.09%	–
Mix	60.19%	–	–	**65.28%**
***DS1*****—100 SAMPLES**				
Not modified	**85.77%**	84.88%	83.35%	84.10%
Linear	83.14%	88.46%	–	–
Non-linear	80.67%	–	89.68%	–
Mix	81.54%	–	–	**90.00%**
***DS2*****—10 SAMPLES**				
Not modified	**53.50%**	51.28%	50.86%	50.48%
Linear	50.59%	56.16%	–	–
Non-linear	50.07%	–	57.35%	–
Mix	49.69%	–	–	**57.53%**
***DS2*****—100 SAMPLES**				
Not modified	**79.54%**	77.37%	76.59%	76.27%
Linear	73.80%	83.77%	–	–
Non-linear	71.46%	–	84.74%	–
Mix	71.31%	–	–	**85.41%**

**Figure 8:**
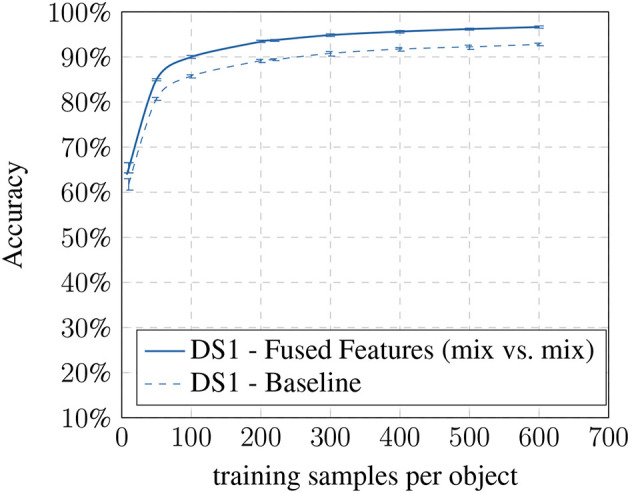
DS1 baseline vs. fused features: accuracy based on training samples per object.

**Figure 9:**
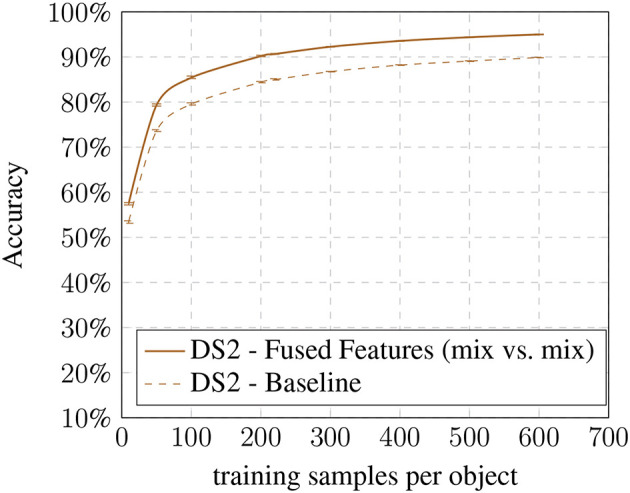
DS2 baseline vs. fused features: accuracy based on training samples per object.

In the last step, we compared the baseline results with our most optimal condition (mix vs. mix) (see Figures 8, 9). Here we show that the improvement is present in all training set sizes and that its effect is not dependent on the number of samples per object.

The results suggest that the additional information of the altered images are beneficial for the recognition process increasing the specificity of the method. While the individual models already achieve an improvement, the biggest gain can be seen while combining both models. However, the improvements come at the cost of a larger feature vector for training which increases the SVM's training time.

## 5. Discussion

As indicated by our previous work (Keller and Lohan, 2016), we have shown that illumination changes have an impact on state-of-the-art object recognition pipelines using DCNNs for feature generation and Multiclass-SVMs for classification. As expected, our results show that using more samples did improve the performance. However, our focus was on training with a small number of training samples to allow for very short interaction periods for data acquisition.

By expanding our work to a second dataset, we have shown that the impact of illumination changes from our first paper is generalizable. Especially, since the iCubWorld Transformation dataset includes many more objects and different types of object manipulations in front of the robot together with higher variability in the background during object presentation.

Training with the artificial illumination models results in a slight drop of performance on the unmodified test sets but results in a major improvement under model-based illumination changes. While these findings are based on models and thus are not directly translatable to natural light changes, it proves that by using data augmentations in the input space the recognition process can become more robust against light changes, resulting in a better generalization of the recognition pipeline.

By adding knowledge about the data generation for data augmentation in the feature space, namely concatenating corresponding feature vectors, we have shown how to make use of our findings to improve the recognition on both datasets which results in a more specialized recognition.

The data augmentations increased the accuracy between 3.54% (*DS1*) and 4.03% (*DS2*) for 10 training samples and between 4.23% (*DS1*) and 5.86% (*DS2*) for 100 training samples. Also, a similar increase is present in all training sample sizes and datasets. The effect might appear as a small improvement only. However, the results show in a systematic way which benefit can be expected from the used models and can serve as a baseline to find better ones. It also shows that the impact increases with more diverse light conditions (*DS1* vs. *DS2*).

Usually, improvements come at a cost. In our case, it is the additional computation with the *mix* condition of the fused feature approach adding the highest amount. It involves generating four more images for the transformations and the feature generation for them. The added computation for the transformations is small compared to the feature generation. Since the images are independent and thus the feature generation can run in parallel the added time cost for this step is small and easily fits into the online learning pipeline. The limiting process is the second step as the SVM has to process a five-times-larger feature vector. While this might render the data augmentation unattractive for large training sample sizes per object, it is still viable for our focus area of small sample sizes. For example, in the case of 28 classes and 100 samples the computation went up from 0.1 to 0.6 s on average still being viable for near real-time purposes.

We believe that improving the recognition of objects with our methods during short interactions (< 1*min*) will enhance the reliability of the overall system and therefore enhance the Human-Robot Interaction and hence, the acceptance of the system.

## 6. Conclusion And Future Work

With our model-based analysis, we showed that changes in illumination affect recognition approaches that use DCNNs to encode features for object recognition. Using data augmentation, we were able to show that a system using DCNNs and SVMs can be modified toward a more robust recognition even though the used DCNN already included an augmentation step toward intensity and color robustness. Additionally, we have demonstrated that using simple brightness change models can help to improve the recognition across all training set sizes.

With our approach, it is easy to adapt existing visual recognition pipelines since only computationally inexpensive data augmentations were used and no modification of either the feature encoder or the classification is needed. Treating the feature encoder as a black-box system allows to compare different networks to the original setup which will be the subject of our future research.

As the next step to improve this approach, we are looking into combining the models and integrating more natural conditions into them. While the artificial models already enhanced the recognition, we believe that with a more realistic representation of natural light changes our approach could be improved. However, the choice of the simple models was due to their low computational cost. This trade-off needs to be taken into account for real-time capable systems like the one we investigation in our paper.

To further improve the acquisition of training data for the objects, the lighting conditions could be artificially altered to generate more diversity that could help the recognition process. When inspecting an object for the first time, a flashlight with known spectral properties or RGB LEDs with defined colors could be used. This might overcome the problem to find a model for real-world light changes as the additional knowledge can be used to inform the data augmentation process.

Additionally, this approach could be used in conjunction with cloud robotics in which multiple robots with different sensors and in different environments could combine their acquired images to cover more diverse illumination settings.

## Data Availability Statement

Publicly available datasets were analyzed in this study. This data can be found here: https://robotology.github.io/iCubWorld.

## Author Contributions

IK and KL contributed to the conception and design of the work. IK organized and performed the analysis, and wrote the first draft of the manuscript. IK and KL wrote the sections of the manuscript. All authors contributed to manuscript revision, read, and approved the submitted version.

### Conflict of Interest

The authors declare that the research was conducted in the absence of any commercial or financial relationships that could be construed as a potential conflict of interest.

## Footnotes

^1^https://www.jibo.com

^2^http://www.bluefrogrobotics.com

^3^http://www.moley.com

^4^https://github.com/robotology/iol

^5^https://github.com/robotology

^6^https://github.com/robotology/himrep
